# E2F1 and p53 Transcription Factors as Accessory Factors for Nucleotide Excision Repair

**DOI:** 10.3390/ijms131013554

**Published:** 2012-10-19

**Authors:** Renier Vélez-Cruz, David G. Johnson

**Affiliations:** Department of Molecular Carcinogenesis, The University of Texas MD Anderson Cancer Center, Science Park, 1801 Park Road 1C, Smithville, TX 78957, USA; E-Mail: rvelez@mdanderson.org

**Keywords:** DNA repair, chromatin, histone acetylation, GCN5, p300

## Abstract

Many of the biochemical details of nucleotide excision repair (NER) have been established using purified proteins and DNA substrates. In cells however, DNA is tightly packaged around histones and other chromatin-associated proteins, which can be an obstacle to efficient repair. Several cooperating mechanisms enhance the efficiency of NER by altering chromatin structure. Interestingly, many of the players involved in modifying chromatin at sites of DNA damage were originally identified as regulators of transcription. These include ATP-dependent chromatin remodelers, histone modifying enzymes and several transcription factors. The p53 and E2F1 transcription factors are well known for their abilities to regulate gene expression in response to DNA damage. This review will highlight the underappreciated, transcription-independent functions of p53 and E2F1 in modifying chromatin structure in response to DNA damage to promote global NER.

## 1. Introduction

Preserving the integrity of genetic information encoded in our genome in order for it to be passed from generation to generation presents a major challenge. This is especially true considering that our genetic material is constantly under attack by a plethora of endogenous and exogenous insults that threaten its integrity. Organisms have developed a variety of DNA repair mechanisms in order to ensure the faithful passing of genetic information. In eukaryotic cells, genomic DNA is wrapped around histones to form nucleosomes, which are further packaged with additional proteins to form higher order chromatin structures. Chromatin not only physically protects the genetic material, but also regulates genetic transactions such as DNA replication, transcription, and repair by controlling access to DNA [1–3]. Specialized enzymes that modulate chromatin structure are therefore of pivotal importance in the control of processes that require access to the genetic material. Regulating access to the genome through chromatin remodeling encompasses a variety of enzymatic activities and processes such as histone acetylation, methylation, phosphorylation, poly(ADP-ribosyl)ation, ubiquitination, and SUMOylation, as well as DNA methylation. These modifications may also lead to nucleosome repositioning, eviction and incorporation [4–7]. While some post-translational modifications can influence the interactions between histones and DNA, others serve as docking sites for accessory factors that aid or impede cellular processes involving the genetic material [8,9]. The importance of enzymes that catalyze chromatin modifications is underscored by the fact that faulty chromatin remodeling, or epigenetic control, can result in a variety of outcomes ranging from premature aging to cancer [10–14].

Given that transcription and DNA repair both involve the manipulation of DNA in the context of chromatin, it is perhaps not surprising that there is considerable overlap between proteins regulating these two processes. Many proteins, such as those in the TFIIH complex, have well-established roles in both transcription and DNA repair. More recently, several other proteins originally identified as repair proteins, including XPA, XPC, XPG, and XPF-ERCC1, have been shown to also function as regulators of transcription [15–17]. This review article will focus on two transcription factors, p53 and E2F1, and their non-transcriptional functions in stimulating nucleotide excision repair (NER) in mammalian cells.

## 2. The Nucleotide Excision Repair Pathway

### 2.1. The Nucleotide Excision Repair Model

The NER pathway is responsible for the removal of a variety of bulky DNA lesions from the genome [18,19]. Overall, NER involves the orchestrated recruitment and action of more than 30 proteins [20,21]. This mechanism is sub-divided into two sub-pathways. Global-genome NER (GG-NER) is responsible for the removal of DNA-distorting lesions from the overall genome. This sub-pathway is initiated by the recognition of a helix-distorting lesion by the XPC-RAD23B protein complex, in some cases with the help of the DDB complex [22]. Among the DNA lesions recognized by this complex are a structurally diverse group including UV-induced photolesions, adducts formed by metabolites of the carcinogen benzo[α]pyrene, and DNA crosslinks formed by the anti-tumor drug cisplatin. The XPC-RAD23B complex stably bound to the DNA lesion allows the recruitment of the TFIIH complex. This ten-subunit complex is endowed with several enzymatic activities such as ATPase/helicases (XPB and XPD) and a cyclin-dependent kinase module (cyclin H, Mat1 and CDK7). Once TFIIH is recruited to the damaged site, its DNA helicases catalyze the opening of the DNA around the lesion, which allows the recruitment of XPA. Replication protein A (RPA) binding to single-stranded DNA further stabilizes this structure and participates in the recruitment of the two DNA endonucleases, XPG and XPF-ERCC1. The activities of these structure-specific endonucleases cut the damaged DNA strand and induce the excision of a 24–30 nucleotide fragment [23,24]. The excision step is followed by the arrival of the DNA re-synthesis machinery: replication factor C (RFC), PCNA, and a DNA polymerase (DNA polymerase δ, ɛ, and κ, depending on cell proliferation status) [25,26]. After the new DNA fragment has been synthesized, the repair process is finished by a DNA ligase (DNA ligase I or III, depending on the cellular proliferation status) [27–30].

The second sub-pathway of NER is known as transcription-coupled NER (TC-NER). TC-NER is responsible for the accelerated removal of transcription-blocking lesions on the transcribed strand of active genes [31,32]. Contrary to GG-NER, TC-NER does not require or depend on the XPC-RAD23B complex for lesion recognition. This pathway is triggered by an RNA polymerase II stalled in front of a blocking DNA lesion. This stalled polymerase induces the recruitment of TC-NER-specific factors CSA and CSB [33–35]. CSB further allows the recruitment of the high mobility group 1 (HMG1) protein, the p300 histone acetyltransferase (HAT) and other accessory factors [33]. The RNA polymerase II-CSB-DNA complex undergoes a structural remodeling that allows the recruitment of TFIIH and XPA, followed by RPA, XPG and XPF-ERCC1, as described for GG-NER [34]. The current model presumes that the final DNA re-synthesis steps are identical for both TC-NER and GG-NER.

The NER pathway is critical for maintaining genome integrity, since failure to remove bulky DNA lesions from the genome is highly mutagenic and cytotoxic. In fact, deficiencies or mutations in NER proteins result in genetic diseases such as xeroderma pigmentosum (XP), trichothiodystrophy (TTD), and Cockayne syndrome (CS) [36]. XP patients with mutations in any of the eight complementation groups (XP-A through G, and XP-V) display a more than 1000-fold increased incidence of UV-induced cancers, sun sensitivity, skin pigmentation abnormalities, and some developmental defects [36,37]. On the other hand, TTD patients with mutations in TTDA/p8, XPB, or XPD display photosensitivity and severe developmental defects, but do not display higher cancer incidence. Similarly, patients with mutations in TC-NER factors (CSA and CSB) develop CS. CS patients do not develop cancers, but develop severe growth defects, neurological abnormalities, mental retardation, sun sensitivity, and early onset aging phenotypes or segmental progeria [38,39]. The high skin cancer incidence of XP patients is attributed to the fact that these patients cannot remove mutagenic DNA lesions induced by UV radiation. On the other hand, the accelerated aging of CS patients is attributed to the fact that they cannot displace an RNA polymerase stalled in front of a DNA lesion, a very potent apoptotic stimulus [40,41]. Additionally, there is a group of patients that display a complex array of severe clinical features including premature aging and cancer. These patients are diagnosed with combined XP/CS and bare mutations in *XPB*, *XPD*, or *XPG* all of which alter the transcriptional activity of TFIIH. Intriguingly, many of the severe clinical features of XP and CS patients cannot be explained solely by a defect in DNA repair and argue for the possibility that NER proteins may play roles in other cellular functions, such as transcription. Furthermore, NER factors were recently shown to be recruited to promoters of RNA polymerase I and II-dependent genes and to help modify chromatin for optimal transcription [15–17,42]. Finally, specific transcriptional networks seem to be altered as a result of mutations in different NER genes [16,43–48].

### 2.2. Nucleotide Excision Repair in Chromatin

Even though we know many of the molecular details of NER, much of the work that has allowed us to acquire our current level of understanding was performed *in vitro* and, importantly, in the absence of chromatin. Early cellular studies showed nucleosomes were re-arranged after UV irradiation and that UV exposure caused an increase in the level of acetylated histones even in the absence of functional NER [49,50]. Furthermore, increasing the levels of acetylated histones by pre-treating cells with *N*-butyrate, a histone deacetylase inhibitor (HDACi), increased the rate of NER [49–51]. Acetylation of lysine residues on histone tails neutralizes the positive charge of these lysines and thus loosens the interaction between histones and the DNA. Importantly, many chromatin-remodeling factors contain bromo domains, which specifically bind to acetylated lysines [8,9]. Thus, histone acetylation in response to UV may also be important for the recruitment of ATP-dependent chromatin remodeling complexes, some of which contain proteins with bromo domains. Not surprisingly, the histone modifying enzymes and chromatin remodeling complexes that have been identified as participating in NER are also important regulators of transcription. For transcription, these factors are often recruited to gene promoters and enhancers through interactions with sequence-specific DNA-binding proteins. However, the mechanisms by which histone modifying enzymes are recruited to sites of DNA damage are less well understood.

## 3. Transcription Factors and the Nucleotide Excision Repair Pathway

### 3.1. p53 as a Chromatin Accessibility Factor for NER

The tumor suppressor p53, also known as the guardian of the genome, is a transcription factor mutated in the majority of human cancers and plays a critical role in the transcriptional response to DNA damage and other types of stress [52]. The p53 protein is phosphorylated by several DNA damage-activated kinases, including the ataxia telangiectasia mutated (ATM), ATM and Rad3-related (ATR), Chk1 and Chk2 kinases, which contributes to p53 stabilization and activation as a transcription factor. Other post-translational modifications, such as acetylation and methylation, also contribute to the regulation of p53 activity in response to DNA damage. The *CDKN1a* gene, which encodes the p21 cyclin-dependent kinase inhibitor, is an important p53 target gene involved in the maintenance of cell cycle checkpoints. Other p53 gene targets are involved in the induction of apoptosis following DNA damage. In addition, p53 can regulate the expression of several genes whose products are involved in DNA repair, including *XPC* and *DDB2*. The transcriptional regulation of these NER genes by p53 was thought to explain the results from early studies demonstrating that cells deficient for p53 are impaired for GG-NER but not TC-NER [53–56].

In addition to regulating the transcription of some DNA repair genes, there is also evidence that p53 plays an important non-transcriptional role in NER as a chromatin accessibility factor. In 2003 Rubbi and Milner showed that a pool of p53 co-localized with sites of NER and that p53 induced chromatin relaxation by recruiting the p300 HAT to sites of DNA damage [57]. This correlated with an increase in global levels of acetylated histone H3 in cells with wild type p53 but not in cells lacking p53. Moreover, it was shown that the inefficient repair of UV-induced damage observed in the absence of p53 could be overcome by treating cells with an HDACi, which increases global levels of histone acetylation. Studies by other groups support the conclusion that p53 enhances chromatin accessibility for NER but whether p53 actually localizes to sites of DNA damage remains controversial [54,58].

Other studies have identified the p33ING1 (inhibitor of growth 1) and p33ING2 proteins as additional chromatin accessibility factors that work with p53 to promote efficient NER. Overexpression of p33ING1 or p33ING2 was shown to enhance the repair of UV-induced DNA damage and this was dependent on p53 [59,60]. On the other hand, depletion of p33ING1 or p33ING2 resulted in impaired removal of photolesions, which could be overcome by pre-treatment with an HDACi [59,61]. Stimulation of NER by p33ING1 and p33ING2 is associated with an increase in global histone H4 acetylation and chromatin relaxation following UV exposure [60,61]. The p33ING1 and p33ING2 proteins do not appear to co-localize with sites of UV-induced DNA damage but they are required for the efficient recruitment of NER factors to damaged sites [60,61].

The GADD45α (growth arrest and DNA damage-inducible) protein may also cooperate with p53 and the p33ING proteins to increase chromatin accessibility for NER. *GADD45A* is a p53 target gene and the GADD45α protein has been shown to bind to p33ING1 [59]. GADD45α-deficient mouse embryonic fibroblasts (MEFs) display slower GG-NER, similarly to p53-deficient MEFs [62]. However, this study did not ask whether the overexpression of GADD45α would enhance repair or whether GADD45α-mediated repair is p53-dependent. GADD45α interacts with UV-damaged nucleosomes, thus suggesting that this protein could play a role in lesion accessibility [62,63]. While the function of GADD45α remains elusive in both repair and transcription, conflicting reports have shown a potential function for this protein in the recruitment of the NER machinery to different promoters to mediate NER-dependent DNA demethylation [64,65].

In summary, several studies have linked p53 and related factors to an increase in global histone acetylation and chromatin relaxation following UV irradiation. This stimulates DNA repair by allowing NER factors to more efficiently access DNA lesions. How this non-transcriptional function of p53 contributes to tumor suppression is unclear.

### 3.2. Transcription-Independent Functions of E2F1 in DNA Repair

The E2F family of transcription factors is composed of 8 members in mammalian cells and, together with the retinoblastoma (RB) tumor suppressor and related proteins, controls the expression of genes involved in DNA synthesis, cell cycle progression and apoptosis [66]. The founding member of the E2F family, E2F1, also plays important roles in the DNA damage response [67]. E2F1 is phosphorylated by the ATM and ATR kinases in response to DNA damage on serine 31, a residue not conserved in other E2F family members [68]. This phosphorylation event helps to stabilize E2F1 and also allows E2F1 to bind the topoisomerase IIβ binding protein 1 (TopBP1) through a phospho-specific interaction requiring the sixth BRCA1 C-terminus (BRCT) domain of TopBP1. TopBP1 binding inhibits the transcriptional activity of E2F1 independent of RB [69,70].

In addition to inhibiting E2F1 transcriptional activity, the phosphorylation of E2F1 by ATM and binding to TopBP1 also recruits E2F1 to sites of DNA double-strand breaks (DSB) [69]. E2F1 foci formation at sites of DSBs does not require a functional DNA-binding domain or the transcriptional activation domain. It was originally thought that TopBP1 binding simply sequesters E2F1 as a mechanism for transcriptional inhibition but more recent data suggests that E2F1 deficiency impairs DSB repair and leads to genomic instability [71]. Whether E2F1 functions to stimulate DSB repair independent of transcriptional regulation is at present unclear.

**Figure 1:**
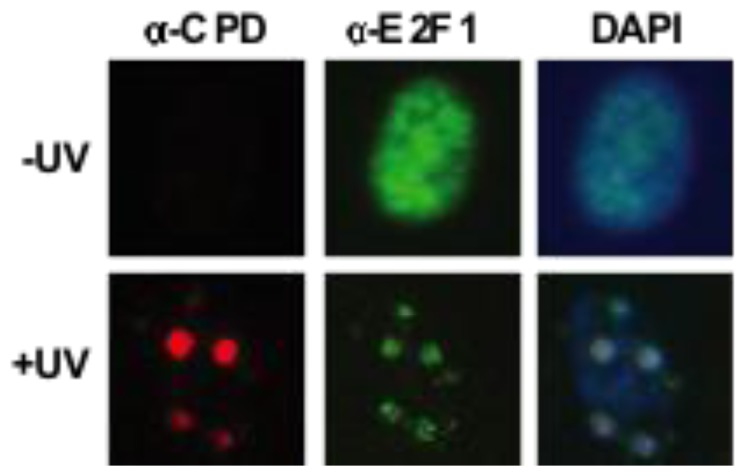
E2F1 accumulates at sites of UV-induced DNA damage. Normal human fibroblasts were untreated (−UV) or locally irradiated with 100 J/m^2^ of UV-C (+UV) through polycarbonate filters with pores of 3 μm as indicated. Cells were fixed 30 min post-irradiation, and stained for CPD photoproducts (red) and E2F1 (green) by indirect immunofluorescence.

Early clues about a role for E2F1 in NER came from the fact that *E2f1*^−/−^ knockout mice are impaired for the repair of UV-induced DNA damage while transgenic mice overexpressing E2F1 in the epidermis display enhanced removal of DNA photoproducts [72]. This effect of E2F1 on NER efficiency correlated with increased sensitivity to UV-induced apoptosis in the absence of E2F1 and resistance to UV-induced apoptosis when E2F1 was overexpressed [72]. Subsequent work from our group showed that E2F1, but not E2F2 or E2F3, accumulates at sites of UV-induced DNA damage [73] (Figure 1). Furthermore, the recruitment of E2F1 to these damaged sites depends on the ATR kinase and serine 31 of E2F1, suggesting that the phosphorylation of E2F1 at serine 31 and binding to TopBP1 may also be involved in the recruitment of E2F1 to sites of UV-induced DNA damage.

Similar to the accessibility defect caused by the lack of p53, the absence of E2F1 impairs the recruitment of NER factors (XPC, XPA, and TFIIH) to sites of damage, and accordingly, depletion of E2F1 by siRNA results in slower removal of UV-induced photolesions. It is important to note that the absence of E2F1 did not alter the expression levels of the NER factors studied. Protein domain analysis of E2F1 concluded that neither the DNA-binding domain nor the transactivation domain are required for the enhancement of NER, providing further evidence that E2F1 stimulates NER through a non-transcriptional mechanism [73].

This non-transcriptional function of E2F1 was subsequently shown to involve the recruitment of the GCN5 HAT to sites of DNA damage [74]. GCN5 co-localizes with sites of UV-induced damage and this is dependent on E2F1. In addition, an increased association between GCN5 and E2F1 was observed in response to UV radiation. E2F1-dependent recruitment of GCN5 to sites of UV damage correlated with an increase in histone H3 lysine 9 (H3K9) acetylation at both damaged sites and globally. In budding yeast, GCN5 is also important for GG-NER by controlling histone H3K9 acetylation [75–77]. Taken together, these findings suggest that like p53, E2F1 also functions as an accessibility factor for NER by recruiting GCN5 to sites of damage and helping to remodel chromatin by promoting H3K9 acetylation and perhaps other histone modifications.

## 4. Conclusion

### 4.1. Unanswered Questions and Future Directions

The regulation of chromatin structure to permit or restrict access to specific DNA sequences is important for all DNA metabolic processes, including transcription and DNA repair. The finding that E2F1 and p53 not only function as sequence-specific transcription factors but also as chromatin accessibility factors for NER adds to the growing list of proteins found to play dual roles in transcription and repair. Given that the primary function of many transcription factors is to recruit chromatin-modifying enzymes to regulate gene expression, it is quite possible that additional transcription factors will also be shown to facilitate NER and other DNA repair mechanisms by modifying chromatin at sites of damage. Indeed, several other transcription factors, in addition to E2F1, have been implicated in the repair of DNA DSBs [78–84].

Here we have highlighted the roles of E2F1 and p53 in regulating histone acetylation in response to UV radiation to promote NER. E2F1 is associated with the recruitment of GCN5 to sites of damage and increased H3K9 acetylation while p53 has been associated with both H3K9 and H4 acetylation and the p300 HAT [57,73,74]. Whether these pathways are complementary to each other or are interrelated remains to be determined. The absence of E2F1 does not affect UV-induced H4K16 acetylation but it is possible that p53 affects E2F1-dependent H3K9 acetylation. This would explain why p53 deficiency impacts both H3K9 and H4 acetylation in response to UV radiation. In addition to these acetylation events, it is also possible that E2F1 and p53 are involved in the recruitment of additional histone-modifying enzymes to regulate chromatin structure and facilitate NER. E2F1 and p53 may also participate in the recruitment of ATP-dependent chromatin remodeling complexes to sites of DNA damage, either directly or indirectly through histone acetylation.

**Figure 2:**
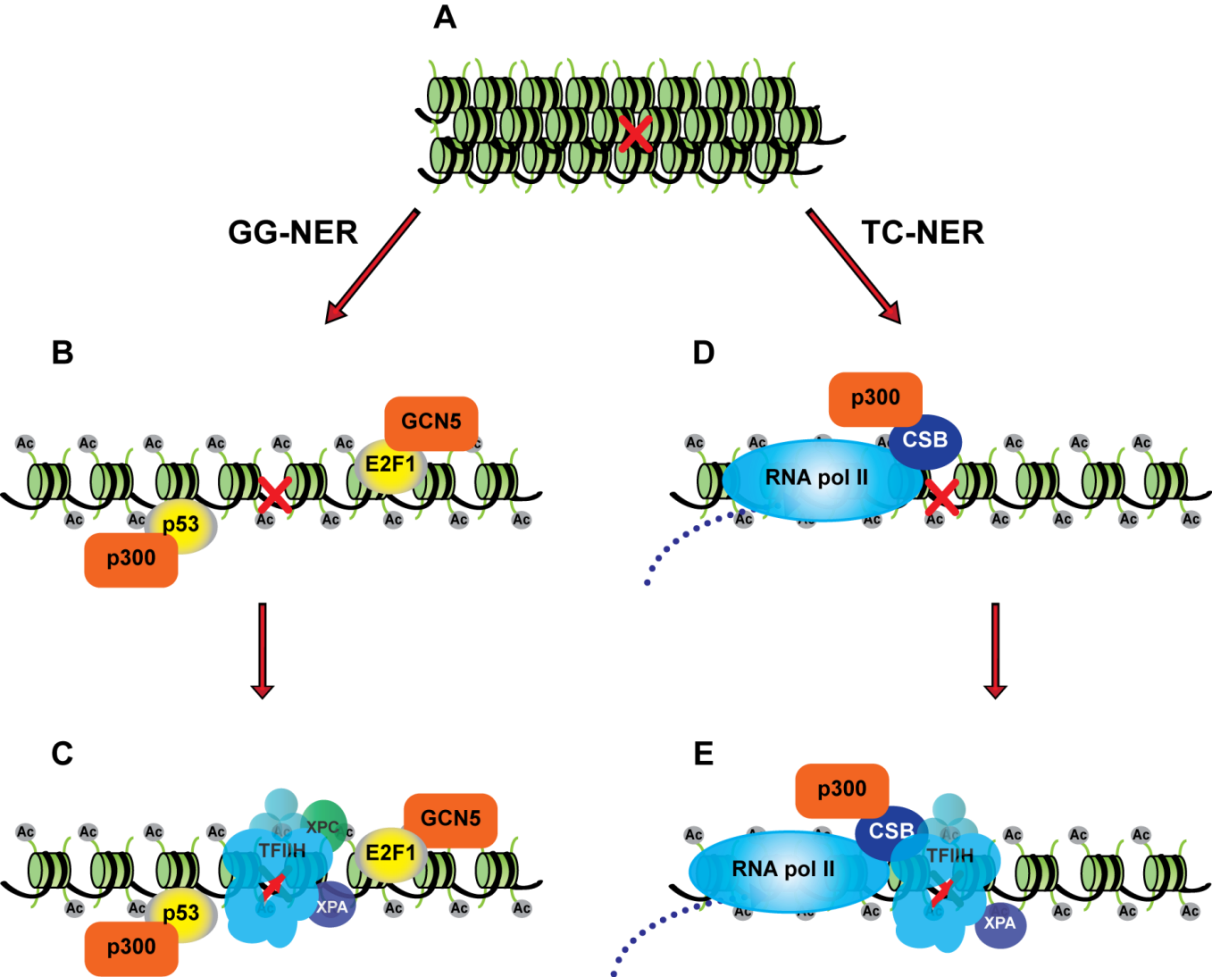
Histone acetylation and chromatin accessibility are important for both global genome NER (GG-NER) and transcription-coupled NER (TC-NER). (**A**) Regions of the genome that are not transcribed are generally in a hypoacetylated state; (**B**) During GG-NER transcription factors p53 and E2F1 are recruited to the damaged sites and facilitate the recruitment of HATs p300 and GCN5, which in turn increase histone H3 and H4 acetylation; (**C**) Hyperacetylation of chromatin at these sites increases access to NER factors (XPC, TFIIH, and XPA) to initiate repair; (**D**) During TC-NER an RNA polymerase II stalled in front of a transcription-blocking lesions recruits CSB, which in turn recruits p300 to the site of a DNA lesion; (**E**) p300 maintains a hyperacetylated chromatin state, thus providing accessibility to the other NER factors (TFIIH, and XPA).

It is interesting to note the importance of histone acetylation for both sub-pathways of NER. During TC-NER, the process of transcription elongation may already lead to increased accessibility of the lesion to the core NER machinery. Moreover, the TC-NER-specific factors, CSA and CSB, function to recruit p300, which likely increases histone acetylation around the damaged site leading to additional alterations in the chromatin environment to aid repair [33]. For GG-NER, E2F1 and p53 appear to be involved in promoting histone acetylation and chromatin relaxation at sites of damage through the recruitment of GCN5 and p300 (Figure 2). These functions for E2F1 and p53 as accessibility factors for GG-NER may be particularly important for repair in heterochromatic regions of the genome.

While relaxing chromatin structure through the activities of transcriptional co-activators (e.g., GCN5, p300) seems to enhance DNA repair, the recruitment of transcriptional co-repressors to sites of damage has also been observed [85,86]. The role that these factors play in the repair process is currently unknown but it has been speculated that they might participate in restoring chromatin structure following the successful removal and repair of the damaged DNA. This would fit the access-repair-restore model envisioned for NER in the context of chromatin [87,88]. It is also possible that these co-repressors function to inhibit transcription at sites of damage to prevent interference with the DNA repair machinery. More recently, it has been proposed that chromatin modifications not only regulate access to DNA damage, but “prime” chromatin for efficient repair [89]. In this model, chromatin serves as a dynamic platform for the recruitment of repair factors as well as proteins involved in DNA damage response signaling. This suggests that chromatin alterations induced by effectors of the DNA damage response at sites of damage may not only stimulate repair, but may also contribute to DNA damage response signaling.

In addition to indirectly promoting DNA repair by regulating chromatin structure, E2F1 and p53 also directly interact with some DNA repair factors [90–93]. For example, E2F1 interacts with NBS1 and this may be involved in regulating the activity of the Mre11-Rad50-NBS1 (MRN) complex at sites of DNA DSBs [71,91]. E2F1 also interacts with DDB2/XPE, a component of a complex that specifically binds damaged DNA and also includes the CUL4A ubiquitin ligase complex [90]. DDB2 was found to directly bind the transcriptional activation domain of E2F1 and to enhance E2F1 transcriptional activity. However, the effect of E2F1 on the ability of DDB2 to stimulate NER through DNA damage recognition has not been examined nor has a potential role for DDB2 in the recruitment of E2F1 to sites of damage been explored. Interestingly, DDB2 was recently shown to promote chromatin relaxation independent of the CUL4A ubiquitin ligase complex [94]. DDB2 has also been shown to interact with the p300 HAT [95] but whether this interaction is involved in E2F1- or p53-dependent histone acetylation and chromatin relaxation in response to UV radiation remains to be determined.

Another unanswered question is how DNA damage induced post-translational modifications to E2F1 and p53 regulate their DNA repair functions [67,96]. It is known that phosphorylation of E2F1 at serine 31 by ATR is required for E2F1 to localize to sites of UV-induced DNA damage and to stimulate NER [73]. E2F1 is also acetylated in response to DNA damage and this enhances E2F1-dependent transcription of the *p73* gene [97–99]. Whether acetylation or other modifications to E2F1 are also involved in regulating its DNA repair activities remains to be determined. Likewise, p53 undergoes extensive post-translational modifications upon DNA damage (phosphorylation, acetylation, methylation, SUMOylation and monoubiquitination) and it remains to be determined how these modifications regulate the DNA repair function of p53.

DNA damage is not only a threat to genomic integrity, but it is also our major weapon against cancer. The majority of current cancer therapies kill cancer cells by damaging their DNA. Therefore, it is of pivotal importance to identify the factors that can confer either sensitivity or resistance to cancer cells in order to improve therapeutic efficacy. The majority of cancers have lost p53 function and many cancers also have deregulated E2F1 function. In theory, cancers that are deficient in p53 and/or E2F1 may be more sensitive to therapies that induce DNA lesions repaired through NER. On the other hand, many cancers display increased levels of E2F1 and this may contribute to resistance against chemotherapeutics such as cisplatin. Future studies should be aimed towards harnessing the potential therapeutic benefits against malignancies that have lost or deregulated the function of these chromatin accessibility factors for DNA repair.
